# Experimental Investigation on the Buckling Capacity of Angle Steel Strengthened at Both Legs Using VaRTM-Processed Unbonded CFRP Laminates

**DOI:** 10.3390/polym13132216

**Published:** 2021-07-05

**Authors:** Fengky Satria Yoresta, Phan Viet Nhut, Daiki Nakamoto, Yukihiro Matsumoto

**Affiliations:** 1 Department of Architecture and Civil Engineering, Toyohashi University of Technology, Toyohashi 441-8580, Aichi, Japan; phan.viet.nhut.yu@tut.jp (P.V.N.); nakamoto.daiki.md@tut.jp (D.N.); y-matsum@ace.tut.ac.jp (Y.M.); 2 Engineering and Building Design Laboratory, Bogor Agricultural University, Bogor 16680, Indonesia; 3 Department of Civil Engineering, The University of Danang—University of Technology and Education, Danang City 550000, Vietnam

**Keywords:** unbonded CFRP, angle steel, buckling, VaRTM, strengthening

## Abstract

Strengthening steel structures by using carbon fiber reinforced polymer (CFRP) laminates showed a growth trend in the last several years. A similar strengthening technique, known as adhesive bonding, has also been adopted. This paper presented a promising alternative for strengthening steel members against buckling by using vacuum-assisted resin transfer molding (VaRTM)-processed unbonded CFRP laminates. A total of thirteen slender angle steel members (L65x6), including two control specimens, were prepared and experimentally tested. The specimens were strengthened only at both legs and were allowed to buckle on their weak axes. The test showed that the unbonded CFRP strengthening successfully increased the buckling capacity of the angle steel. The strengthening effect ranged from 7.12% to 69.13%, depending on various parameters (i.e., number of CFRP layers, CFRP length, and angle steel’s slenderness ratio). Flexural stiffness of the CFRP governed the failure modes in terms of location of plastic hinge and direction of buckling curvature.

## 1. Introduction

The use of CFRP as a strengthening material for concrete structures has gained wide acceptance worldwide due to its excellent properties [1,2,3,4,5], i.e., light weight, excellent fatigue behavior, resistance to corrosion, high strength-to-weight ratio, and easy installation. However, this has not entirely been the case for steel structures. Research and development on the use of CFRP for strengthening steel structures is still limited. A method that has been developed is the same as that used for strengthening concrete structures, which involves attaching the CFRP to the steel surface [6,7,8,9,10] and is well known as an externally bonded strengthening technique.

Indeed, the externally bonded strengthening technique is effective for enhancing the performance of steel structures. It has been convincingly proven that this technique is able to increase: the compression capacity of short steel members [11,12,13], flexural capacity of steel beams [14,15,16,17,18], torsional capacity of steel members [19,20], load bearing capacity of steel shear wall [21,22,23], tensile capacity of steel plates [24,25,26,27,28], and also buckling capacity of long steel columns [29,30,31]. However, this strengthening technique is considered to have drawbacks. The most critical issue is bond strength between CFRP and steel substrate [32,33]. Debonding failure between steel and CFRP has occurred in many investigations [34,35,36,37,38], often before CFRP can provide its maximum contribution as a strengthening material. In order to obtain a better bond between CFRP and steel, the bonded strengthening technique requires proper steel surface treatments (for example, sandblasting, hand grinding, and/or grit blasting) prior to installation of CFRP. The surface treatment activity becomes much more complicated and difficult when it is applied to existing steel buildings because of the uncertainty and complexity of the real conditions in field. Different types of steel surface treatments will also effect to the bonding strength of CFRP to steel [39]. Moreover, the choice of one of the treatments cannot also completely ensure that the bond strength of CFRP to steel will be uniform because there can be differences in the quality of the steel surface after treatment. This is largely because the workers who perform the treatment often have different levels of skill [40].

Even supposing that steel surface treatments can be performed very well, the bond strength between steel and CFRP remains prone to deterioration due to inevitable environmental exposure. Experimental research has proven that the load-carrying capacity of the CFRP/steel adhesive-bonded joints is significantly reduced at temperatures around and above the glass transition temperature (*T*_g_) of the adhesive [41,42]. The CFRP/steel bond strength also decreases in subzero temperatures (−40 °C, using MBrace Saturant) [43]. In addition, moisture absorption can affect the performance of CFRP composites [44,45].

In this paper, unbonded CFRP is proposed as an alternative to avoid the problems arising in the bonding method. The unbonded CFRP is used to strengthen angle steel against buckling. The unbonded CFRP strengthening method eliminates the steel surface preparation process required by the adhesive-bonding method, because CFRP is not adhesively attached onto steel substrate [46]. An unbonded layer will exist between these two materials as a separator. This means unbonded CFRP strengthening can be applied faster and easier on-site. Moreover, as it does not depend on the bond between steel and CFRP, the unbonded CFRP strengthening method is likelier to be used for a longer period of time compared to the adhesive-bonded strengthening. There will be no concerns about the degradation of strengthening performance due to environmental exposure. The performance of unbonded CFRP strengthening will depend only on the flexural rigidity of the CFRP laminates.

## 2. Materials and Methods

### 2.1. Materials

**Figure 1:**
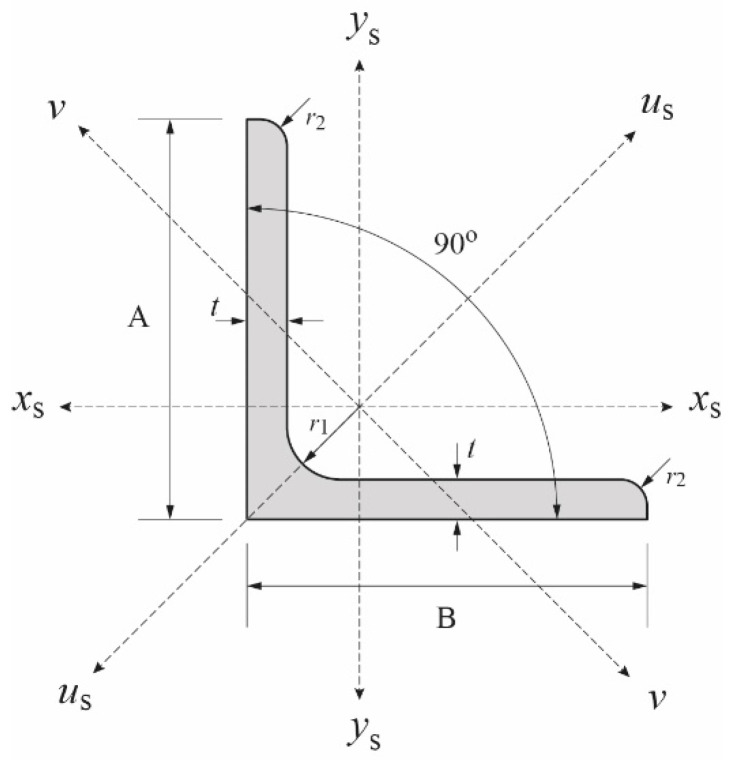
Angle steel cross-section.

Angle steel L-65x6 is used as the main material in this experimental program. The steel has a grade SS400 according to JIS G3101:2020 (Japanese Industrial Standard). Figure 1 shows cross section of the steel with values of each symbol given in Table 1. It should be noted that measured wall thickness *t*_a_ is 5.56 mm, which is slightly smaller than the nominal wall thickness *t* given in the table. Material properties of the steel angle, which are also presented in the table, are obtained from the inspection certificate issued by the manufacturer. There was no tensile coupon test conducted in this study.

**Figure 2:**
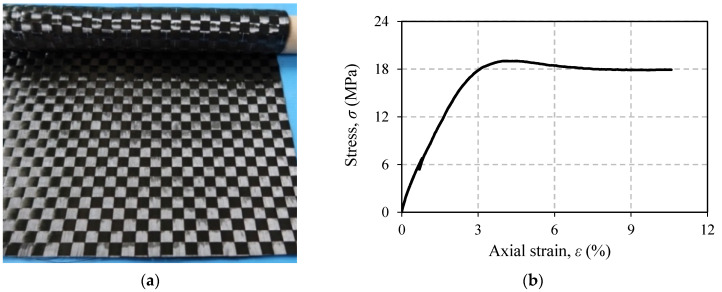
Materials for CFRP: (**a**) carbon fiber BT70-20; (**b**) typical stress–strain curve of the epoxy adhesive.

The CFRP laminates are made of bidirectional carbon fiber (termed as BT70-20) which is a product of Toray Industries, Inc., Tokyo, Japan. In one sheet, this material has two directions of fiber which are perpendicular to each other (Figure 2a). With this configuration, a laminate with the same properties in both directions of the weave (0° and 90°) can easily be produced. This is the main reason for choosing this material in this study. Table 2 shows properties and characteristics of carbon fiber BT70-20. All the data given are based on the manufacturer’s data sheet. In addition to carbon fiber, the adhesive used is epoxy resin (a product of Konishi Co., Ltd., Osaka, Japan). Modulus of elasticity, Poisson’s ratio, and tensile strength of this material are 1.285 GPa, 0.46, and 18.50 MPa, respectively. These data are average values obtained from tensile tests of six specimens. Figure 2b shows a representation of the stress–strain curve of the epoxy resin.

### 2.2. Description of Specimens

**Figure 3:**
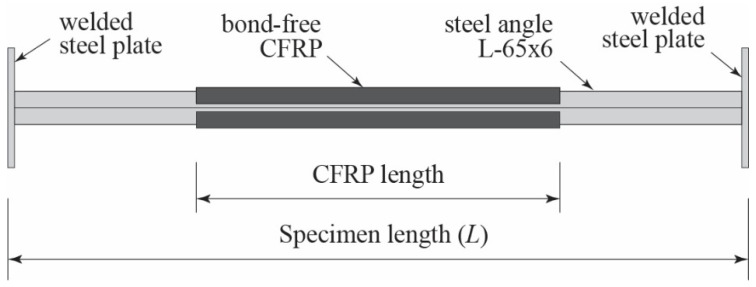
Strengthening scheme for the test specimen.

**Figure 4:**
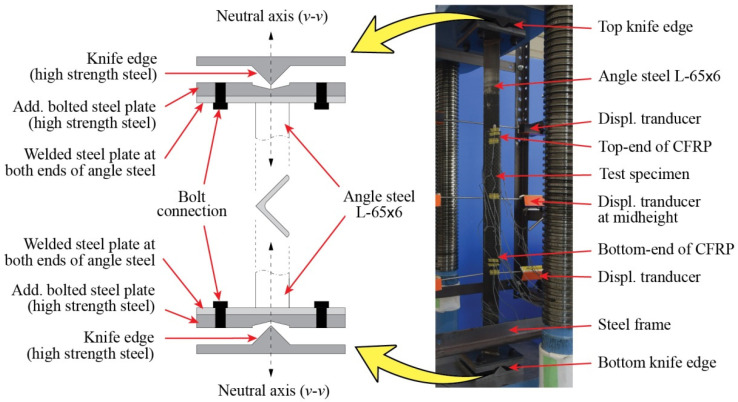
Test setup.

The specimens tested in this study consist of two groups of angle steel with different slenderness ratios (λ). Each group has one control specimen that is not strengthened with CFRP. Specimens of Group A (λ = 128.4) are divided into two different CFRP lengths, i.e., 1000 mm and 500 mm, while specimens of Group B (λ = 97.02) have CFRP length of 500 mm. The number of CFRP layers in strengthened specimens is varied to further investigate the effect of strengthening. The CFRP is positioned in the middle of the steel, as shown in Figure 3. The total number of specimens tested is thirteen, and their detailed parameters are given in Table 3. As shown in the table, specimens are labeled for easy identification. The first combination of ‘letter-number’ describes angle steel and its buckling length, the second combination is strengthening length, and the last combination indicates number of CFRP layers. For instance, A16S10L25 identifies a specimen with 1636 mm buckling length, 1000 mm CFRP length, and 25 CFRP layers. Here, buckling length is a total of specimen length *L* (Figure 3) and thickness of additional steel plates at both ends of specimen (Figure 4).

### 2.3. Specimens Preparation

**Figure 5:**
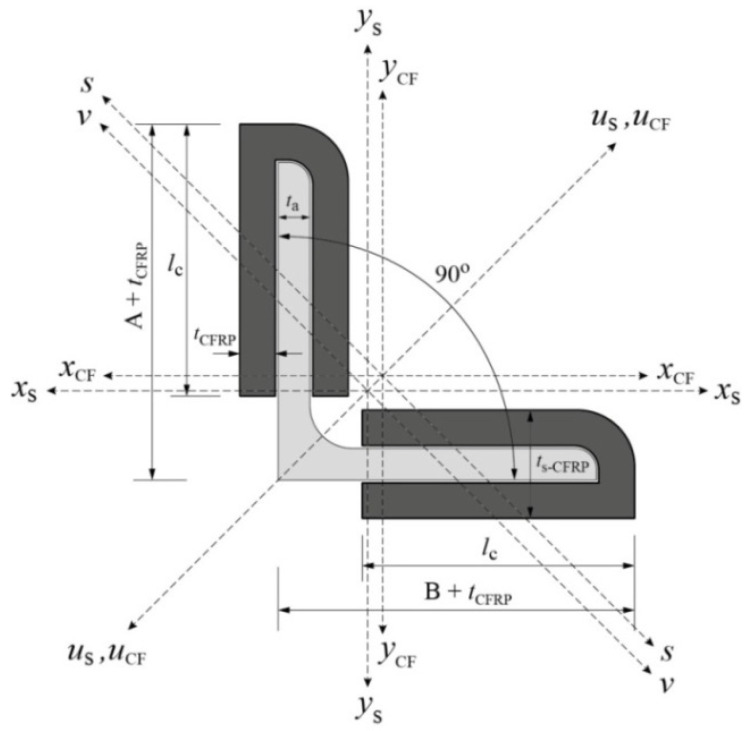
Strengthening scheme for the test specimen.

**Figure 6:**
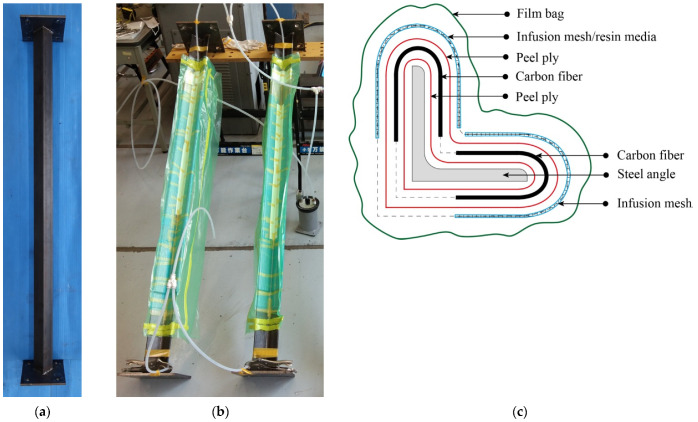
Specimen preparation: (**a**) angle steel before strengthening; (**b**) specimens ready for impregnation process; (**c**) cross section of specimen during molding process.

Both ends of steel angle are welded onto steel plates which are used for the installation of knife edges later on. Welding is carried out so that weak axis of the angle steel coincides with center line of the steel plate. The unbonded CFRPs in all strengthened specimens are fabricated through a process of vacuum-assisted resin transfer molding (VaRTM). The process begins by wrapping the steel angles in the mid-span position (strengthening zone) using one layer of peel ply. The wrapping process is not preceded by any treatment on the steel surface. The carbon fiber with a predetermined number of layers is then installed following the peel ply. The fiber, at 0°, is positioned in the same direction as the longitudinal axis of the steel angle. The carbon fiber is only assigned to both legs of the angle steel, not to cover the entire cross section (see Figure 5). Afterwards, an additional layer of peel ply is used to cover the carbon fiber. Infusion meshes (resin media) are attached after the peel ply to facilitate resin transfer during the impregnation process. A vacuum bag with a strong gum tape connection is applied at the same time as the installation hose in both injection and vacuuming sides. The next process is to impregnate resin by suction. The resin impregnation process is carried out by tilting the specimens. This aims to decrease the risk of air bubbles trapped inside the mold. After CFRP is molded, specimens are cured for at least one week before being tested. Figure 6 shows the process of specimen preparation.

### 2.4. Test Setup and Instrumentation

**Figure 7:**
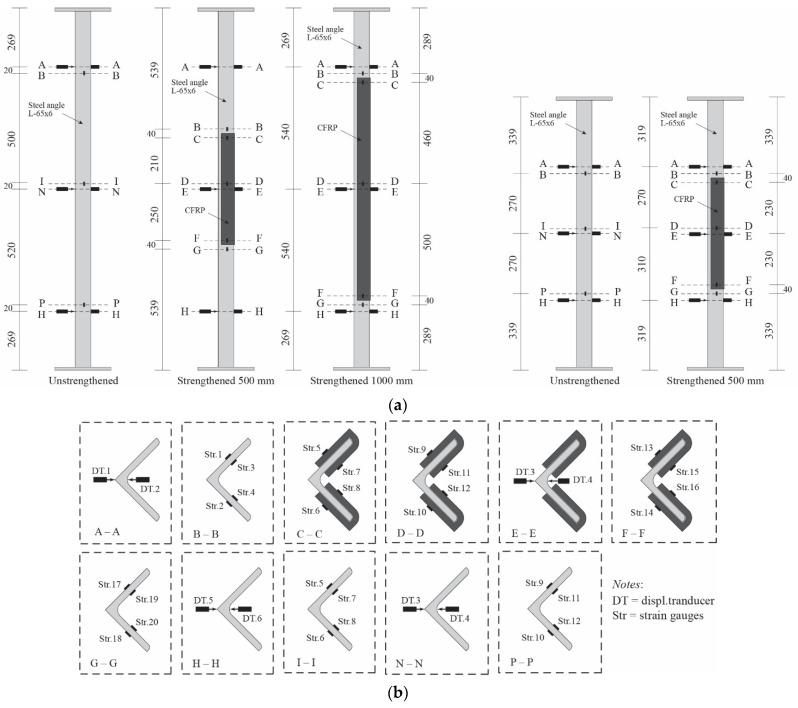
Position of displacement transducers and strain gauges: (**a**) overall position; (**b**) detail in cross section.

All the strengthened and control specimens are tested under compression load using a 2000 kN capacity of Maekawa testing machine, as shown in Figure 4. The specimens are in pinned-end conditions and designed to buckle on their weak axis (*v*–*v*) only (Figure 1). To achieve these conditions, a knife edge is installed in both ends of specimens (Figure 4) where it must also coincide with the weak axis. During the test, the applied load and axial displacement are measured by a load cell within the machine. A total of 20 strain gauges are installed, scattered along the strengthened specimen to measure its longitudinal strain response. Meanwhile, only 12 strain gauges are used for control specimen. Out-of-plane lateral displacement is measured by displacement transducers. The lateral displacement is measured at three different locations: around the bottom end of CFRP, mid-height of specimen, and around the top end of CFRP. Figure 7 shows the detailed position of strain gauges and transducers on both control and strengthened specimens.

## 3. Results and Discussion

Table 4 summarizes the results of the investigation in this study, including maximum load capacity, CFRP thickness, and fiber content of the laminates. Also presented in Table 4 is the strengthening effect due to the application of unbonded CFRP. The strengthening effect is given as the ratio of the difference of maximum load capacity of specimens (between control and strengthened specimens) and maximum load capacity of the appropriate control specimen (A16S00L00 or A12S00L00). Based on Table 4, it is clear that unbonded CFRP provides considerable positive strengthening effects. The greatest effect was found in specimen A16S10L35 (69.13%), whereas specimen A16S05L18 experienced the least strengthening effect (7.123%). Generally, the positive strengthening effect and its variation in values are attributed to a delay in the overall buckling. These delays are due to the different values of flexural stiffness of the unbonded CFRP which exist in each specimen. The difference in CFRP flexural stiffness is defined by the application of different parameters, e.g., number of CFRP layers and CFRP length. Another factor that exists is difference in fiber content of CFRP in each specimen, as given in Table 4. The fiber content also contributes to flexural stiffness of CFRP, although not to the same extent as number of CFRP layers and/or CFRP length, by creating an elastic modulus of the laminates. Theoretically, the higher the fiber content, the higher the elastic modulus of CFRP, and thus the greater the stiffness.

The fiber content, as presented in Table 4, is a ratio between total thickness of carbon fiber used (number of layers × 0.112 mm) and thickness of CFRP. The thickness of CFRP (*t*_CF_) is calculated by involving the wall thickness of the specimen at the strengthened part (*t*_tot_) and the wall thickness of the angle steel (*t*_a_ = 5.56 mm), namely *t*_CF_ = 0.5 (*t*_tot_ − *t*_a_). It is evident from Table 4 that CFRP has stable and higher fiber content (>50%, with a coefficient of variation of 2.30%). Therefore, in addition to being highly expected, this confirms the advantages of the VaRTM process for producing CFRP laminates over the other techniques (e.g., hand-layup) [47].

### 3.1. Load–Displacement Response

**Figure 8:**
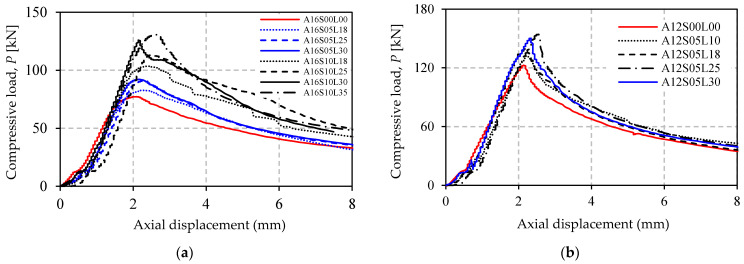
Load–axial displacement response of specimens: (**a**) Group A; (**b**) Group B.

Figure 8a,b shows the load versus axial-displacement response of all the specimens in Group A and B. It can be seen from the figure that the curves provide a steep initial response before reaching maximum load. After maximum load, the axial displacement increases rapidly as the compressive load gradually decreases. Figure 8 graphically proves that the presence of unbonded CFRP increases the maximum load, but it does not affect the initial axial stiffness of the strengthened specimens. The slope of the curves remains unchanged compared to the control specimen. This is reasonable as the CFRP is not bonded to the steel surface, which means that elastic behavior of the structure will not be affected.

**Figure 9:**
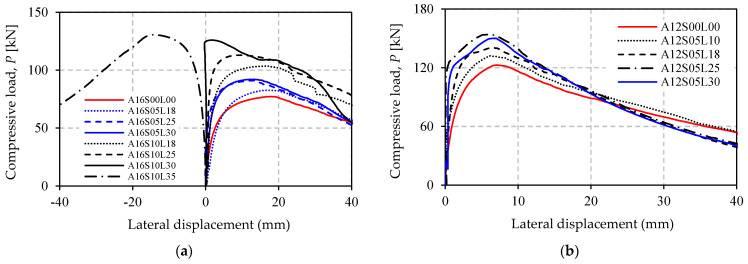
Load–lateral displacement response at mid-height of specimens: (**a**) Group A; (**b**) Group B.

The load–lateral displacement responses at mid-height of the specimens are provided in Figure 9a,b. The curves show a steep response before overall buckling occurs. Afterward, lateral displacement increases rapidly. The displacement curve of A16S10L35 shows a negative value, indicating that this specimen buckles in the opposite direction to the others. It is clear from Figure 9 that unbonded CFRP reduces the lateral deflection.

### 3.2. Effect of Number of CFRP Layers

**Figure 10:**
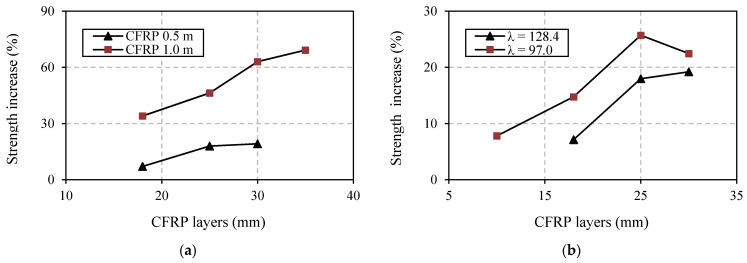
Effect of several parameters: (**a**) effect of CFRP length (λ = 128.4); (**b**) effect of angle steel’s slenderness ratio (CFRP length = 500 mm).

Increasing the number of CFRP layer gives a positive effect in enhancing the buckling strength of the angle steel. This is confirmed by Figure 10 and Table 4, and can also be seen in Figure 8 and Figure 9. The strength increase in the specimens in Group A ranged from 7.123% to 69.13%, but in Group B the increases only varied between 7.742% and 25.67%. The relationship between increasing strength and increasing the number of CFRP layers is experienced by all strengthened specimens with appropriate CFRP length in both Group A and B. However, the exception is specimen A12S05L30, which has 30 CFRP layers. The strength increase in this specimen (22.45%) is not larger than that of specimen A12S05L25 (25.71%), which only has 25 CFRP layers. The reason for this is most likely the higher imperfection of specimen A12S05L30. The imperfection is indeed not measured in this study, but it is reflected in the load–lateral displacement curve of this specimen (see Figure 9a). The curve is less vertical (before reaching ultimate load) compared to that of specimen A12S05L25.

### 3.3. Effect of CFRP Length

The effect of CFRP length is well displayed through comparison of specimens in Group A (see Table 4) where two types of CFRP length are investigated, 500 mm and 1000 mm. For easy visualization, the comparison is also presented in Figure 10a. It is clear from Figure 10a and Table 4 that strength increase is higher for specimens with longer CFRP. The difference in strength increase between specimen A16S10L18 (34.03%) and specimen A16S05L18 (7.12%), both with 18 layers of CFRP, is 34.04% − 7.12% = 26.92%. This value is almost unchanged with the difference in strength increase in other specimens (A16S10L25 and A16S05L25), where the number of CFRP layers is increased to 25 (46.28% − 17.97% = 28.31%). However, for specimens with more than 25 layers and 1000 mm of CFRP length (e.g., A16S10L30) there tends to be a greater strength increase compared to specimens with 500 mm CFRP length (e.g., A16S05L30).

### 3.4. Effect of Different Slenderness of Steel

Figure 10b shows the effect of different angle steel’s slenderness ratio (λ) on the strength increase in the strengthened specimens. A detailed percentage of the increases is presented in Table 4. Figure 10b and Table 4 show that a higher strength increase belongs to specimens with a smaller angle steel’s slenderness ratio. This condition is clearly demonstrated by all comparable specimens (same as number of CFRP layers) between Group A and B having CFRP length of 500 mm. Compared to a specimen with a larger λ (A16S05L18, 18 CFRP layers), a higher strength increase occurs in a specimen with a smaller λ (A12S05L10), even though fewer layers of CFRP are used (10 layers).

### 3.5. Failure Modes

**Figure 11:**
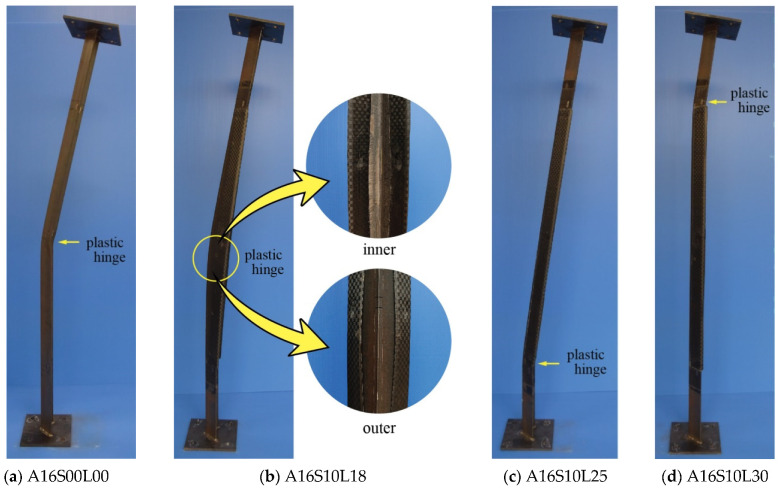

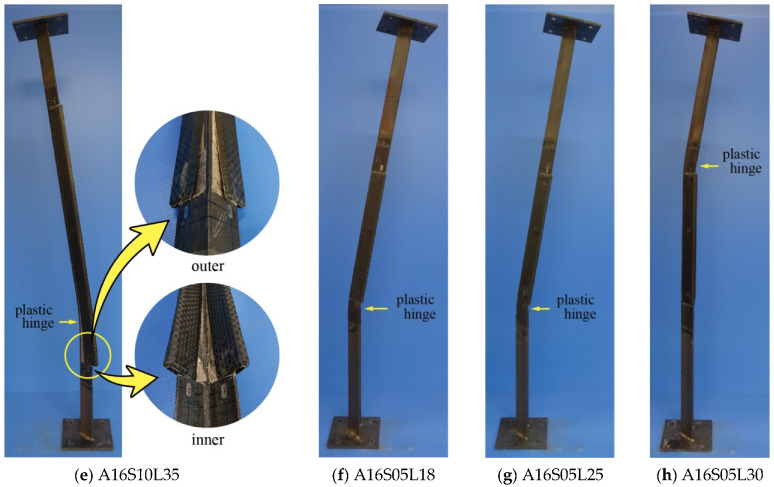
Failure mode of specimens: (**a**) A16S00L00; (**b**) A16S10L18; (**c**) A16S10L25; (**d**) A16S10L30; (**e**) A16S10L35; (**f**) A16S05L18; (**g**) A16S05L25; (**h**) A16S15L30.

**Figure 12:**
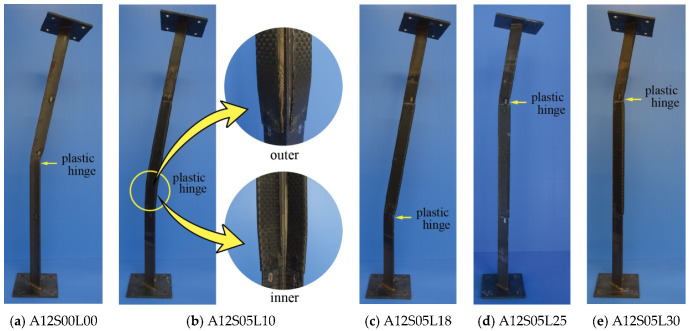
Failure mode of specimens: (**a**) A12S00L00; (**b**) A12S05L10; (**c**) A12S05L18; (**d**) A12S05L25; (**e**) A12S05L30.

All control specimens undergo failure due to overall buckling of pinned-end columns, as shown in Figure 11a and Figure 12a. Plastic hinge is confirmed at mid-height of the specimens. For the unbonded CFRP strengthened angle steel, three different failure modes can be observed. The first mode of failure is that the plastic hinge is located around the edge of the CFRP (outside CFRP strengthening zone), not at mid-height of the specimens, as shown in Figure 11c,d,f–h) and Figure 12c–e. The buckling curvatures are similar to each other, toward the inner side of the angle steel. In this case, the CFRP laminates can perform their duty as a stiffener without suffering damage. In the second mode, the specimen fails when the plastic hinge lies within the strengthening zone, as shown in Figure 11b and Figure 12b. The direction of the curvature remains the same as the former failure mode, i.e., to the inner side of the angle steel. This mode of failure occurs when the number of CFRP layers is lower (A16S10L18 and A12S05L10), meaning that the CFRP does not have the appropriate stiffness to overcome overall buckling of the angle steel. Thus, the CFRP at the outer side of the angle steel peels off and the CFRP at the inner side of the angle steel is under compression, which can be damaged when excessive load is applied (see Figure 11b and Figure 12b).

The last failure mode occurs when the plastic hinge is within the CFRP strengthening zone but the buckling curvature occurs towards the outer side of the angle steel, as experienced by specimen A16S10L35 (Figure 11e). This type of failure mode tends to occur as the number of CFRP layers increases. This trend can be easily seen in specimens belonging to Group A which have a CFRP length of 1000 mm (Figure 9a). It is clear from Figure 9a that the lateral deflection curve (before reaching maximum load) becomes steeper as the number of CFRP layers increases from 18 (A16S10L18) to 25 (A16S10L25). It is only within the extreme condition of the use of 30 CFRP layers that the specimen curve (A16S10L30) becomes perfectly vertical. Beyond this, a larger number of layers makes the curve response change to a negative value (specimen A16S10L35). This means that the buckling curvature occurs in the opposite direction (Figure 11e). According to the authors, the reason behind this failure mode is that, in addition to the increase in the CFRP stiffness due to the increase in the number of CFRP layers, loading eccentricity (in terms of the centroid of the CFRP’s cross section) also increases. The larger number of CFRP layers leads to a larger change in the centroid of the CFRP cross section, but the position of the applied load remains unchanged on the weak axis of the angle steel cross section.

### 3.6. Strain Response

**Figure 13:**
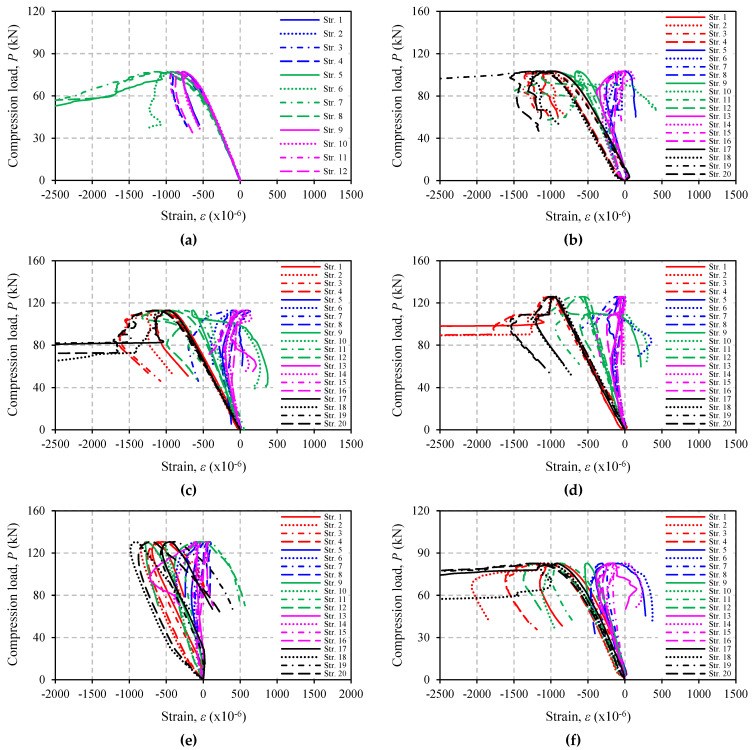

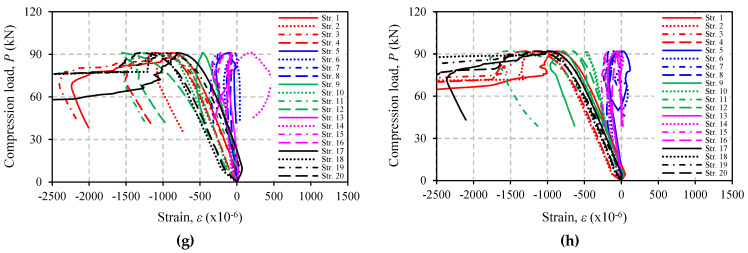
Failure mode of specimens: (**a**) A16S00L00; (**b**) A16S10L18; (**c**) A16S10L25; (**d**) A16S10L30; (**e**) A16S10L35; (**f**) A16S05L18; (**g**) A16S05L25; (**h**) A16S05L30.

**Figure 14:**
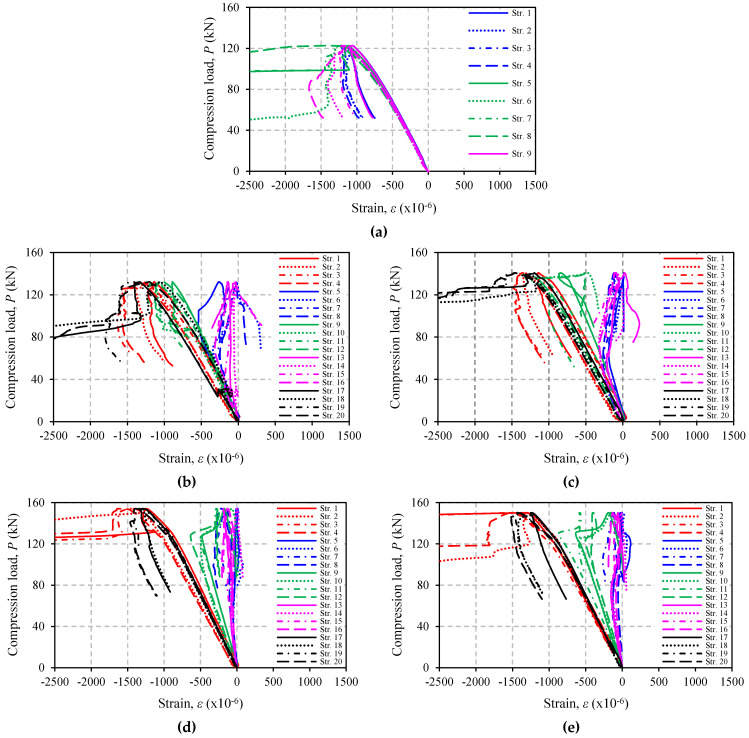
Failure mode of specimens: (**a**) A12S00L00; (**b**) A12S05L10; (**c**) A12S05L18; (**d**) A12S05L25; (**e**) A12S05L30.

Figure 13 and Figure 14 show the load versus longitudinal strain response of all specimens tested in this study. It is confirmed by Figure 13a and Figure 14a (control specimens) that only compressive strain at mid-height of the specimens continues to develop and reaches the angle steel yield strain at about 0.16%. This finding corresponds to the failure modes previously discussed. For strengthened specimens, the strain on CFRP also increases, but the rate of increase at the end of CFRP is smaller than that at the middle, and it only occurs up to a certain load (the load where strain stops developing or returns to zero), e.g., in specimen A12S05L18 (see Figure 14c for easy visualization, the load is around 60 kN). In other specimens, the strains in CFRP may not develop at all or develop only up to a very small load, for example, specimens A12S05L25 or A12S05L30 (Figure 14d,e). These two specimens have higher CFRP stiffness as a result of many layers of CFRP and shorter CFRP length. In some other specimens (mainly in Group A), the load at which the strain stops developing may not be easily identified. The increase in compressive strains on CFRP in all of the strengthened specimens indicates the existence of a minor bond of CFRP to steel. This is highly possible because during the VaRTM process, the resin will also impregnate into the peel ply.

## 4. Conclusions

This paper investigates the use of unbonded CFRP laminates, as an alternative to adhesive-bonding CFRP, for strengthening angle steel members against buckling. The CFRP is fabricated directly onto the surface of the steel members, using the VaRTM technique, and is adjusted to cover both legs of the angel steel only. Based on the experimental investigation, the following findings can be drawn:

1. Unbonded CFRP strengthening can successfully increase the buckling capacity of the angle steel. The strengthening effect varies, and the maximum load increase is 69.13%;
2. The buckling capacity of angle steel increases with increasing number of CFRP layers and CFRP length. In addition, to strengthen without changing CFRP length, the increase in buckling capacity is greater for specimens with shorter angle steel (smaller angle steel’s slenderness ratio);
3. Based on the position of the plastic hinge, failure modes of the unbonded CFRP strengthened specimens can be divided into two: plastic hinge at around the end of CFRP (outside CFRP strengthening zone), and plastic hinge within the CFRP strengthening zone. Both failure modes are affected by flexural stiffness of the CFRP laminates;
4. The VaRTM process is the best alternative for making CFRP. The CFRP fabricated through the VaRTM process has a higher fiber volume content of around 60%.

It should be noted that angle steel is often used as a lateral resisting element in building structures, so it is very susceptible to buckling failure and needs to be strengthened. Therefore, in conjunction with this research program, the authors also demonstrate the potential of unbonded CFRP strengthening for real-world applications.

## Figures and Tables

**Table 1:** Parameter and properties of the steel angle.

Designation	Cross-Section Dimension (mm)	Yield Stress (MPa)	Tensile Strength (MPa)	Elastic Modulus (MPa)
L-65x6	A = B = 65; *t* = 6.0; *r*_1_ = 8.5; and *r*_2_ = 4.0	333	448	205,000

**Table 2:** Carbon fiber.

Type	Orientation of Fiber	Tensile Strength (MPa)	Elastic Modulus (MPa)	Thickness (mm)
BT70-20	0^o^	2900	230,000	0.056
	90^o^	2900	230,000	0.056

**Table 3:** Description of the test specimens.

Specimen Name	Number of CFRP Layers (ply)	CFRP Length (mm)	Buckling Length (mm)	Angle Steel’s Slenderness Ratio
*Group A*				
A16S00L00	-	-	1636	128.4
A16S10L18	18	1000	1636	128.4
A16S10L25	25	1000	1636	128.4
A16S10L30	30	1000	1636	128.4
A16S10L35	35	1000	1636	128.4
A16S05L18	18	500	1636	128.4
A16S05L25	25	500	1636	128.4
A16S05L30	30	500	1636	128.4
*Group B*				
A12S00L00	-	-	1236	97.02
A12S05L10	10	500	1236	97.02
A12S05L18	18	500	1236	97.02
A12S05L25	25	500	1236	97.02
A12S05L30	30	500	1236	97.02

**Table 4:** Summary of test result.

Specimen Name	CFRP Thickness (mm)	Fiber Content (%)	Max. Load (kN)	Strengthening Effect (%)
*Group A*				
A16S00L00	-	-	77.22	-
A16S10L18	3.34	60.35	103.5	34.03
A16S10L25	4.58	61.15	113.0	46.34
A16S10L30	5.50	61.11	125.9	63.04
A16S10L35	6.38	61.47	130.6	69.13
A16S05L18	3.39	59.44	82.72	7.123
A16S05L25	4.65	60.22	91.10	17.97
A16S05L30	5.62	59.84	92.05	19.20
*Group B*				
A12S00L00	-	-	122.7	-
A12S05L10	1.97	56.76	132.2	7.742
A12S05L18	3.30	61.17	140.8	14.75
A12S05L25	4.78	58.59	154.2	25.67
A12S05L30	5.60	59.96	150.2	22.41

## Data Availability

The data required to reproduce these findings cannot be shared at this time, as they also form part of an ongoing study.
